# Case of Enteric Fever with Bicytopenia

**DOI:** 10.7759/cureus.1910

**Published:** 2017-12-06

**Authors:** Madeeha Subhan, Waleed Sadiq

**Affiliations:** 1 Capital Hospital Islamabad, Ayub Teaching Hospital, Abbottabad; 2 Internal Medicine, Shifa International Hospital

**Keywords:** enteric fever, hematology, infectious diseases

## Abstract

Infectious diseases are one of the major causes of morbidity and mortality in developing countries. Typhoid has its own contribution to the disease burden, especially in Pakistan and other tropical countries. Herein, we present a case of enteric fever with a rare presentation. Our patient is a 20-year-old man who gradually developed high-grade fever lasting seven days associated with rigors and chills. No additional accompanying systemic signs helped to localize the infection. After extensive laboratory testing, his typhoid serology was positive along with leukocytopenia and thrombocytopenia. Typhoid fever is typically associated with either diarrhea or constipation and sphygmothermic dissociation (Faget’s sign); our patient did not have these symptoms or signs. As leukocytopenia and thrombocytopenia contribute to mortality and complications, it was necessary to monitor the patient accordingly.

## Introduction

Typhoid, also known as enteric fever, is a multisystem illness caused most commonly by Salmonella typhi, with symptoms ranging from mild to severe. This disease is typically spread by unsafe water and sanitation in impoverished areas [1]. Despite the use of antibiotics, it remains endemic in developing countries [2]. The usual symptoms are sustained fever, chills, and abdominal pain. Additionally, infected individuals may experience rash, nausea, anorexia, diarrhea or constipation, headache, and/or reduced level of consciousness [3].

## Case presentation

A previously healthy 20-year-old man presented to the hospital with a one-week history of remittent high-grade fever (103°F) associated with rigors and chills. The fever cycled daily; it was gradual in onset, settling with antipyretics, but was followed by profuse sweating. Nausea was present, but no episode of vomiting, diarrhea or constipation occurred. The patient provided a history of two weeks with a sore throat. He is a non-alcoholic and nonsmoker with access to clean water. He denied any recent history of contacts with the sick, travel, mosquito bites, weight loss, headache, body aches or pains, bleeding, urinary complaints, or jaundice. On examination, he was febrile and tachycardic, had pale skin, and appeared toxic. Results from his neurological and cardiovascular exams were normal. On abdominal exam, bowel sounds were audible. The abdomen was soft and non-tender, and no hepatosplenomegaly was appreciated. Since there was no localization of infection on history or physical exam, an extensive laboratory workup was done. Only the results of the Typhidot® (immunoglobulin G and M) (Reszon Diagnostics International Sdn. Bhd., Selangor, Malaysia) were positive. Additionally, the test results indicated a very low platelet count of 5,000/µL and a white blood cell (WBC) count of 3,700/µL; it is unusual for enteric fever to cause thrombocytopenia and leukopenia. He was started on antipyretics, antibiotics (azithromycin and meropenem), a proton pump inhibitor, platelet replacement therapy, and packed red blood cells. After treatment, his platelet levels improved to 25,300/µL, and his hemoglobin improved from 7.4 g/dL to 11.3 g/dL in seven days. His complete blood count and complete metabolic profile are shown in Tables 1-2 below.

**Table 1:** Comparison of Complete Blood Picture at the Time of Presentation and One week After Treatment

	At time of presentation	One week after treatment
Red blood cell count	4.2 x 10^6^/µL	3.6 x 10^6^/µL
White blood cell count	3,700/µL	7,000/µL
Platelet count	5,000/µL	25,300/µL
Hemoglobin	7.4 g/dL	11.3 g/d

**Table 2:** Comparison of Complete Metabolic Profile at the Time of Presentation and One Week After Treatment

	At the time of presentation	One week after treatment
Potassium	3.8 mmol/L	4.1 mmol/L
Sodium	134 mmol/L	139 mmol/L
Bilirubin	0.7 mg/dL	0.7 mg/dl
Alanine aminotransferase	36 U/L	35 U/L
Alkaline phosphatase	119 U/L	98 U/L
Creatinine	0.1 mg/dL	0.7 mg/dL
Urea	14 mg/dL	15 mg/dL
Glucose	118 mg/dL	99 mg/dl

## Discussion

The annual incidence of typhoid fever was documented as 209/100,000 persons from 1972 to 1973 [4]. Another study showed an incidence of 48/100,000 persons from 1978 to 1981 [5]. Where typhoid is endemic, patients admitted to hospitals are usually between 5 to 25 years of age [3]. As typhoid develops, the predominant symptom is fever. The patient usually has anorexia, nausea, and vomiting; in severe cases, constipation or bloody diarrhea will occur. Mild normocytic normochromic anemia is a common finding [6]. Only 2% of patients had isolated thrombocytopenia, as reported by Serefhanoglu, et al. [7]. In a study conducted by Qamar, et al., cytopenia was found in 98% of enteric fever patients; amongst these, anemia was present in 61.3%, leucopenia in 52%, thrombocytopenia in 39.3%, and thrombocytopenia alone in 2% [8]. A study by Abro, et al. in 2009 found anemia in 61.3%, thrombocytopenia in 40%, leukocytosis in 10.6%, and leucopenia in 4% of the cases reviewed [9]. Another study found leukopenia in 21%, leukocytosis in 2.46%, anemia in 10%, thrombocytopenia in 14.8%, and pancytopenia in 4.93% of the cases reviewed [10]. Our patient had bicytopenia with an extremely low hemoglobin (7.4 g/dL) and platelet count (i.e., 5,000 /µL).

## Conclusions

Typhoid fever remains a significant health burden, especially in low- and middle-income countries. Despite the availability of more recent data on enteric fever, additional research is needed in many regions. Enteric fever has variable hematological manifestation, such as anemia and bicytopenia. Early diagnosis and treatment may decrease morbidity and mortality.
